# Does work have to be so painful? A review of the literature examining the effects of fibromyalgia on the working experience from the patient perspective

**DOI:** 10.1080/24740527.2020.1820858

**Published:** 2020-12-03

**Authors:** K. Mukhida, W. Carroll, R. Arseneault

**Affiliations:** a Pain Management Unit, Department of Anesthesiology, Pain Management and Perioperative Medicine, Dalhousie University, Halifax, Nova Scotia, Canada; b Department of Management, Sobey School of Business, Saint Mary’s University, Halifax, Nova Scotia, Canada

**Keywords:** fibromyalgia, work, accommodations

## Abstract

**Background:**

Chronic pain conditions, such as fibromyalgia, adversely affect individuals’ abilities to work.

**Aim:**

The aim of this study was to examine, from the perspective of patients, the effects that fibromyalgia symptoms had on their ability to work, the challenges that they encountered in the workplace that did not foster their continued employment, and the types of modifications to their work or workplace that they thought would facilitate their productivity and ability to work.

**Methods:**

A scoping review method, applying techniques of systematic review, was used to conduct a research synthesis of the literature regarding fibromyalgia and work that looked at this issue from the patient perspective.

**Results:**

A variety of themes emerged from the analysis and could be broadly categorized into (1) the work experience was a challenging one with which to cope; (2) relationships were strained at work; (3) clinical symptoms had repercussions on subjects’ attitudes toward work and the relation to life outside of work; and (4) a variety of possible solutions were considered to help subjects better cope with fibromyalgia and work.

**Conclusions:**

Strategies that potentially could foster continued employment of patients with fibromyalgia include those at the micro, meso, and macro levels. Health care providers can support patients’ employment goals by collaborating with patients and their employers, dispelling stigma regarding the illness, and providing practical and specific advice regarding workplace accommodations.

## RÉSUMÉ

**Contexte**: Les douleurs chroniques, comme la fibromyalgie, affectent négativement la capacité des individus à travailler.

**Objectif**: Le but de cette étude était d’examiner, du point de vue des patients, les effets des symptômes de la fibromyalgie sur leur capacité à travailler, les défis qu’ils ont rencontrés dans leur lieu de travail qui n’a pas favorisé leur maintien dans l’emploi, et les types de modifications à leur travail ou lieu de travail qui, selon eux, faciliteraient leur productivité et leur capacité à travailler.

**Méthodes**: Une méthode d’examen de la portée, appliquant des techniques de revue systématique, a été utilisée pour effectuer une synthèse de la littérature concernant la fibromyalgie et le travail qui examinait ce problème du point de vue du patient.

**Résultats**: Divers thèmes ont émergé de l’analyse et pourraient être globalement classés comme suit : (1) l’expérience de travail était difficile à gérer ; (2) les relations étaient tendues au travail ; (3) les symptômes cliniques ont eu des répercussions sur les attitudes des sujets envers le travail et leur rapport à la vie en dehors du travail ; et (4) diverses solutions possibles ont été envisagées pour aider les sujets à mieux faire face à la fibromyalgie au travail.

**Conclusions**: Les stratégies susceptibles de favoriser l’emploi continu des patients atteints de fibromyalgie se situent aux niveaux micro, méso et macro. Les prestataires de soins de santé peuvent soutenir les objectifs d’emploi des patients en collaborant avec eux et leurs employeurs, afin de dissiper la stigmatisation qui entoure la maladie.

## Introduction

A common chronic pain condition that disproportionately affects women, fibromyalgia has pervasive influences on patients’ quality of life and so, not surprising, has detrimental effects on their ability to work.^1^ Studies have consistently shown that a significant proportion of patients with fibromyalgia have difficulties remaining employed. For example, questionnaire-based studies have found that between 35% and 50% of patients with fibromyalgia were not working.^2–5^ A survey of patients being followed at tertiary-level rheumatology clinics found that though 50% of patients with fibromyalgia were employed, only 15% had full-time employment.^4^ Of those employed, 80% planned on continuing to work but 34% of those who were unemployed could not imagine themselves working.^4^ A cohort study involving patients with fibromyalgia, chronic fatigue syndrome, or multiple chemical sensitivities seen at the Environmental Health Clinic at the Women’s College Hospital in Toronto found that 68.8% of patients had to stop working because of their clinical condition.^6^ Similar to the findings of Al-Allaf,^7^ Guymer and colleagues’^3^ survey of members of the Fibromyalgia Support Network of Western Australia was revealing in showing that employment status changed over the course of the disease. In that study, 54.2% and 21.5% of patients were working either full- or part-time, respectively, at the time of symptom onset.^3^ Less than 5 years later, only 15.6% were working full-time and 44.8% were not working at all.^3^

The ability of patients with fibromyalgia to work normal hours has also been shown to be reduced. Patients with fibromyalgia were found to miss more days of work than their colleagues without fibromyalgia.^8,9^ Choy et al.^10^ found that almost half of all patients who were able to continue working for the preceding 12 months still missed at least 10 days of work. About one-third of patients followed by Martinez et al.^11^ reduced their work hours in order to accommodate for their symptoms. Fibromyalgia was the most common reason for sick leave among a cohort of 635 long-term sick-leavers studied in a National Insurance survey.^12,13^

As a consequence of these difficulties with employment, a significant proportion of patients with fibromyalgia receive financial subsidies. Studies have shown that between 34.8% to 55% of patients received some form of financial compensation, such as Social Security Disability.^3,11,14,15^ Overall disability is higher among workers with fibromyalgia compared to those without: patients with fibromyalgia reported worse function, more days in bed, and more healthy years of life lost compared to controls.^16^

Part of the reason for the increased employment challenges faced by patients with fibromyalgia is related to the pursuit of clinical care related to the fibromyalgia diagnosis and treatment of its symptoms. Studies from North America and Europe demonstrate that patients with fibromyalgia spend considerable time attending health care appointments.^17,18^ A study of patients in France and Germany found that they attended between 2.9 and 4.9 medical clinic visits within the 3 months preceding the study’s onset and between 11.6 and 19.6 visits per year.^19^ A study based out of seven American health care centers found that patients with fibromyalgia were hospitalized as inpatients once every 3 years and made approximately 10 outpatient visits per year.^20^ In a Canadian setting, the mean number of visits to family physicians and specialists within the year preceding the study onset was 10.7 and 13.7, respectively, which exceeded the number of visits that patients without fibromyalgia made (2.9 and 0.79, respectively).^6^ Choy et al.^10^ found that 83% of patients in their study sample of 800 patients in Europe, Mexico, and South Korea had visited their physicians at least once per month, and 41% were making at least 2 visits per month.

The overall costs of fibromyalgia incorporate expenses related to such health care utilization and significant indirect costs as well.^21^ Studies have shown that these costs for patients with fibromyalgia exceed those for the general population severalfold. For example, a review of a United States health insurance database found that the direct costs related to fibromyalgia were three times higher (US$9573 versus US$3291) than for patients without fibromyalgia.^22^ The costs of hospitalization alone for patients with fibromyalgia in the United States was estimated to exceed US$1 billion annually.^23^ In Canada, private insurance companies were found to spend US$200 million annually on fibromyalgia-related health care expenses.^24^ Direct costs related to prescription medications, physician visits, and allied health care personnel visits amounted to US$951 in 3 months in a recent Québec study.^25^ In addition to these direct costs of health care are those indirect costs related to absenteeism and presenteeism and associated lost productivity. Lacasse et al.^25^ acknowledge that quantifying the magnitude of these costs is challenging. Nevertheless, estimates of these costs in European and Japanese studies suggest that such indirect costs can exceed direct ones from anywhere from two to six times.^19,26^

Results revealing the significant burden that fibromyalgia places on patients’ abilities to remain gainfully employed and its associated costs are particularly troubling given the important role that work has on well-being. The development of a chronic illness, like chronic pain, becomes, for many patients, a “life-altering experience” because it can result in “shrinking lifeworlds,” “constrained daily paths,” “altered social careers and networks,” and “socio-spatial imprisonment and/or isolation.”^27(p1107)^ Employment is associated with both economical and psychological well-being for patients with chronic pain.^28–32^ For example, for patients with fibromyalgia, persistent employment translated to reduced poverty.^33^ The ability and/or opportunity to continue working also contribute to improved “status,” the sense of having a “mission in life,” and a “link to society.”^34(p567)^ As one patient has explained: “Yesterday I laid in bed and didn’t feel very well, and today I laid in bed and didn’t feel very well. I mean, there are no highlights in that. You must of course have moments like that, but then it feels so good to work, because you come back to work and there is someone who has missed you when you were gone.”^35(p1625)^ To continue working also provides a sense of normalcy: “I guess you feel a little humiliated if you don’t have a job, and I therefore feel so sorry for all unemployed people because it feels like you’re a second class citizen.”^35(p1625)^

For patients with fibromyalgia, continued employment has also been associated with improved health outcomes. For example, both Rakovski et al.^5^ and Palstam et al.^36^ found that patients with fibromyalgia had improved health outcomes if they continued working. Women with fibromyalgia who worked demonstrated better ratings with regards to pain severity, fatigue, stiffness, depression, and physical-related quality of life compared to nonworking women with fibromyalgia in Palstam et al.’s^36^ cross-sectional study.

The aim of this study was to examine, from the perspective of patients: the effects that fibromyalgia symptoms had on their ability to work; the challenges that they encountered in the workplace that did not foster their continued employment; and the types of modifications to their work or workplace that they thought would facilitate their productivity and ability to work.

Because health care practitioners are frequently consulted by patients, insurance providers, and employers to provide opinions regarding the influence of fibromyalgia symptoms on patients’ abilities to work and on workplace accommodation issues, this study suggests ways in which such practitioners can aid patients with fibromyalgia with their employment goals.

## Methods

Using a scoping review method^37^ as a guide, in combination with techniques of systematic review,^38^ a comprehensive search was undertaken of the following academic databases from 1982 to July 2020: PubMed EBSCOhost, Premier EconLit, PsycARTICLES, EBSCO Business Source Premier, and PsycINFO. The following search terms were used: “fibromyalgia” and “work.” These terms were purposefully kept broad to increase the probability that any relevant studies were found. The search was limited to articles published in English. The titles and abstracts of all retrieved studies were reviewed. The reference lists of studies were also reviewed for other potential studies. Google Scholar was also used to aid these searches. Studies were excluded if they did not deal specifically with fibromyalgia and if they did not include any reference to its effects on individuals’ work experiences or abilities to work. This study expands upon the review by Palstam and Mannerkorpi.^39^ In addition to updating the search, a broader search strategy was employed. Instead of including cross-sectional, case–control, and epidemiological studies, we focused only on those studies that considered employment issues from the perspective of patients with fibromyalgia. Thus, our study reviews an additional 17 qualitative studies and 7 questionnaire studies. Whereas Palstam and Mannerkorpi^39^ focus on what patients with fibromyalgia can do to modify their work ability, we additionally describe the implications of those findings specifically on how they inform better strategies that physicians can use to help their patients with employment issues.

### Exclusion Criteria

Studies that did not meet the inclusion criteria included those that, for example, (i) dealt only with the indirect or direct costs of fibromyalgia or health care utilization^7,9,14,17,19,21–23,25,26,40–45^; (2) dealt only with fibromyalgia symptoms^6,46^; (3) were not specific enough to fibromyalgia^47–49^; (4) were not qualitative^50,51^; (5) were reviews^33,34,39,52–60^; (6) were commentaries^61–64^; (7) were related to fibromyalgia pathophysiology,^65^ psychophysiology,^66^ or psychobiological models^67^; (8) dealt with disability- or activity-related issues but not in a manner that specifically addressed patients’ subjective attitudes toward work^68–76^; (9) related to fibromyalgia etiology with respect to work^77,78^; (10) studied the effects of therapies^79–84^; (11) looked at fibromyalgia epidemiology^85,86^; or (12) examined medicolegal issues.^87^

## Results

The search strategy yielded 1526 studies. After review of the titles and abstracts as well as other studies found, 118 studies were further evaluated for relevance to the current topic. A total of 37 studies met the inclusion criteria and were analyzed (Tables 1 and 2).

**Table 1.** Qualitative studies reviewed

Study	n	Patients	Recruitment	Setting	Analysis	Findings
Acker^88^	10	>18 years	Wait list of a rheumatology practice	Boston, MA	Grounded theory	Physicians’ roles in reducing stigma about FM
		Women				
Asbring^89^	13	Women	Hospital units	Sweden	Grounded theory	FM changes identity with respect to work life
Bossema et al.^90^	10	Women	Announcements on the website of the Dutch FM Association	Netherlands	Review of interview statements	Suitable work from the perspective of patients with FM is that which is paced to allow them to perform the task well and with satisfaction, is supported by management and coworkers, and enables sufficient energy for leisure and home activities
Dennis et al.^91^	20	Self-declared diagnosis	Online support group and “real-world” support group	United Kingdom	Interpretative phenomenological analysis	FM has varying effects on the work experience, ranging from job loss to support from Occupational Health and coworkers
Ferguson^92^	13	Men >1 year since FM diagnosis Currently employed or employed for at least 6 of last 12 months	Online support group	Bloomington, IN	Phenomenological analysis	FM symptoms affected career trajectories and patients’ abilities to work to their perceived potential Occupational disclosure of illness could have beneficial or deleterious effects
Gauer^93^	14	25–50 years Diagnosed by physician at least 1 year prior Currently full-time in the United States At least college education	Word of mouth, via call for research participants at FM clinics and at National FM Association website	United States	Thematic analysis	Symptom severity, variability, and unpredictability affect work ability FM symptoms cause frustration with work ability, fear of losing work, and pondering of finding work that fits their limitations Exposure of illness at work is feared to potentially jeopardize work opportunities Education of coworkers about FM would help work environment
Hallberg and Carlsson^94^	22	22-60 years Women Diagnosed according to ACR criteria	Inpatients at national insurance hospital or outpatients at chronic pain unit; strategic recruitment	Sweden	Grounded theory	Work experiences were unsatisfying
Juuso et al.^95^	15	38-64 years Women Diagnosed according to ACR criteria	Rehabilitation center patients and members of the Association for Rheumatism and FM	Sweden	Phenomenological hermeneutic method of interpretation	Hardship associated with FM symptoms and others’ doubts regarding the reality of their pain experience influence attitudes toward work
Juuso et al.^96^	15	38-64 years Women Diagnosed according to ACR criteria	Rehabilitation center patients and members of the Association for Rheumatism and FM	Sweden	Phenomenological hermeneutic method of interpretation	Work is valued but symptoms may preclude ability to perform work Support at work is needed to facilitate ongoing employment
Liedberg and Henriksson^4^	48	Women Working or non-working Physician-verified diagnosis	Respondents to mailed questionnaire	Sweden	Thematic analysis	FM-related limitations in work ability as well as the work-related abilities to make accommodations influence patients’ abilities to continue working
Liedberg et al.^97^	94	Women Newly diagnosed by rheumatologists	Patients at a university referral center and an outpatient clinic	Sweden and United States	Thematic analysis	FM affected adversely affected friendships at work
Löfgren et al.^98^	12	Had participated in a rehabilitation program for FM Had working capacity Currently working	Mailed questionnaire	Sweden	Thematic analysis of interviews, focus group discussions and diaries	Patients with FM use a variety of action-oriented strategies to stay working
Mannerkorpi and Gard^99^	27	33-62 years Diagnosed by physician Currently working	Invited by mail	Sweden	Thematic analysis	The ability for work tasks and environments to be modified influence patients’ abilities to work
Oldfield et al.^100^	9	>18 years Diagnosed by physician	Invited via listservs, posters, newsletters, networking	Toronto	Thematic analysis	Disclosure about FM occurs in a variety of ways in the workplace Patients are selective about how much information is revealed and to whom
Oldfield et al.^101^	9	>18 years Women Diagnosed by physician	Invited via listservs, posters, newsletters, networking	Toronto	Thematic analysis	Working is a way of being “normal” and not “giv[ing] in” to their illness
Palstam et al.^35^	27	30-63 years Currently working Diagnosed by physician	Invited via mail	Sweden	Content analysis	Patients value working Social support within and outside of the workplace facilitated continued employment
Paulson et al.^102^	14	41-56 years Men Married to or living with female partner Diagnosed by physician	Recruited from rheumatology clinic	Sweden	Phenomenological heuristic interpretation	Work provides meaningful tasks Not being able to work allowed freedom from explaining their symptoms and constraints
Paxman^103^	50	Self-declared diagnosis	Randomly selected narratives from The Experience Project website	United States	Thematic analysis	FM symptoms often require curtailment of work hours
Sallinen et al.^104^	20	34-65 years Women	Participants in an FM rehabilitation course	Finland	Thematic analysis	FM symptom fluctuation contributes to difficulties with employment
Sallinen and Mengshoel^105^	5	Men Self-declared diagnosis	Invitations via voluntary organizations	Sweden	Thematic analysis	Work lives are adjusted to accommodate for FM symptoms by changing work-related tasks, reducing work hours, and preserving work ability by decreasing time spent on leisure activities
Söderberg et al.^106^	50	Women Diagnosed according to ACR criteria	Patients in rehabilitation center and rheumatology clinic	Sweden	Phenomenological heuristic interpretation	Fatigue adversely affected work ability
Söderberg et al.^107^	5	Men Married to spouse with FM	Husbands of members of the Rheumatology Association	Sweden	Thematic analysis	Spouse’s inability to work or reduced work adversely affected household finances

MA = Massachusetts, FM = fibromyalgia, IN = Indiana, ACR = American College of Rheumatology.

**Table 2.** Questionnaire studies reviewed

Study	n	Patients	Recruitment	Setting	Analysis	Findings
Blom et al.^108^	64	>18 years Employed Diagnosed by physician	Hospital via rheumatologists	Netherlands	Illness Invalidation Inventory Illness Cognition Questionnaire Bern Embitterment Inventory	Patients with FM experience embitterment resulting from invalidation and helplessness Education of people in the workplace could target invalidation
Boehm et al.^109^	157	>18 years Stated that had been diagnosed with FM by physician	Attendees at FM patient conference	Israel	Fibromyalgia Impact Questionnaire Short-Form Health Survey Modified Social Capital Questionnaire COPE-Multidimensional Coping Inventory	Bonding social capital contributes to improved functioning and quality of life
Choy et al.^10^	800	Women and men Stated that had been diagnosed with FM by physician	Identified by physicians who were treating patients with FM	France, Italy, Germany, Spain, Mexico, South Korea, Netherlands, United Kingdom	Summary of survey responses	FM affected the ability of patients to work and advance their careers
Collado et al.^110^	325	16-64 years old Diagnosed by physician	Epidemiological study with probability sampling procedure	Spain	Summary of survey responses	FM affected patients’ abilities to remain employed, perform work, cope with usual work hours; not all patients were able to obtain accommodations
Côté^111^	3	Diagnosed by physician	Advertisement in newspaper	Quebec	Fibromyalgia Impact Questionnaire Physical Work Performance Evaluation	A work-related rehabilitation and reintegration program helps patients’ work capacities and functional status
Guymer et al.^3^	287	Self-declared diagnosis	Invitation to members of FM Support Network of Western Australia via email and newsletter	Australia	Summary of survey responses	Work ability is affected from the time of FM symptom onset Work disability may be prevented by timely diagnosis and treatment
Liedberg and Björk^112^	362	Self-declared diagnosis Members of the FM chapter of the Swedish Rheumatism Association	Mailed questionnaire	Sweden	Summary of survey responses	Education of employers about the effects of FM on work ability can promote continued employment
Linder et al.^12^	635	Long-term sick leavers	Patients seen in a unit specializing in multidisciplinary investigation of those on long-term sick leave	Sweden	Comprehensive Psychopathological Rating Scale Short-Form 36	Patients with psychiatric-somatic comorbidities would benefit from rehabilitation programs that take them into account
Linder et al.^13^	92	Women Long-term sick leavers Diagnosed by physician	Patients seen in a unit specializing in multidisciplinary investigation of those on long-term sick leave	Sweden	Comprehensive Psychopathological Rating Scale Short-Form 36	FM symptoms affect patients’ work abilities
Martin et al.^113^	80	18-65 years Diagnosed by physician	Rheumatology clinic database and clinic referrals	Birmingham, AL	NEO Five Factor Inventory Coping Strategies Questionnaire Sickness Impact Profile	Teaching patients methods to cope with psychological distress and pain may reduce distress at work
Palstam et al.^36^	129	18-60 years Women Diagnosed by physician	Patients seen in three primary health centers by systematic search of patient journals	Sweden	FM Impact Questionnaire Medical Outcome Study Social Support Survey Hospital Anxiety and Depression Scale Multidimensional Fatigue Inventory	Patients who were able to work reported better health than non-patients
Palstam et al.^114^	73	22-63 years Diagnosed according to ACR criteria	Advertisements in local newspaper	Sweden	FM Impact Questionnaire Fear Avoidance Beliefs Questionnaire Physical Activity Index	Patients with FM perceive their physical exertion at work to be greater than that perceived by their coworkers
Rakovski et al.^5^	1702	Self-declared diagnosis	National FM Association	United States	Summary of survey responses	Patients with FM may benefit from work accommodations to facilitate continued employment
Rivera et al.^115^	301	>18 years Women and men Diagnosed according to ACR criteria	Patients seen as outpatients in rheumatology clinics	Spain	FM Impact Questionnaire FM Health Assessment Questionnaire	Temporary work disability in patients with FM is associated with sedentary work, symptom severity, self-perceived health, and health care resource utilization
White et al.^16^	100	Diagnosis by physician	Telephone survey	London, ON	FM Impact Questionnaire	FM symptoms result in work disability

FM = fibromyalgia, AL = Alabama, ON = Ontario.

A variety of themes emerged from review of the results of the qualitative studies that met the inclusion criteria. These are broadly categorized into themes of (1) the work experience being a challenging one with which to cope; (2) relationships being strained at work; (3) repercussions of fibromyalgia on subjects’ attitudes toward work and its relation to life outside of work; and (4) subjects’ perspectives on the possible solutions to helping them better able to cope with fibromyalgia and work.

### Clinical Symptoms Cause Hardship at Work

One of the most common themes among the qualitative studies that met the inclusion criteria was the hardship at work that they experienced due to their clinical symptoms. Both pain and fatigue were the most commonly described symptoms that contributed to difficulties that they experienced. Work sometimes remained unfinished due to increases in the severity of pain.^92^ One patient described the process of walking down a hallway as being of sufficient physical activity as to cause exhaustion.^92^ Another described the fatigue as being akin to the sensation as though “you’re a balloon and someone’s taken all the air out of you.”^92(p33)^ Clinical symptoms caused subjects to adjust the manner in which they approached their work tasks, including the need for pacing themselves and prioritizing work assignments.^89^ Part of the sense of hardship was not only due to the accentuation of the physical aspects of the jobs that the clinical symptoms caused but also related to the jobs becoming seemingly more challenging to accomplish as previous senses of competence and self-confidence were “demolished.”^89(p313)^

Subjects expressed fear related to their job situation as a result of the recognition that job hardship had affected their ability to work as they had prior to symptom onset or in comparison to colleagues. For example, some subjects feared that they would lose their jobs.^91^ Others feared that colleagues or employers would discover that they had a chronic pain problem.^91^ Palstam et al.^114^ studied the role that perceived exertion played in influencing attitudes toward work among women with fibromyalgia. Despite physical activity not differing between women workers with fibromyalgia and those without, perceived exertion at work was higher and physical capacity was lower among those women with fibromyalgia. This was associated with factors related to fear avoidance work beliefs and anxiety. The study’s authors suggested that the subjects with fibromyalgia may have been anxious about the manner in which their current work habits could affect their future ability to work, the need for sick leave in the future, and “physical overload” that would preclude them from being able to manage their clinical symptoms and work over the long term.^114(p778)^

Subjects reported that their fibromyalgia symptoms prevented them from “living up to [their] work potential.”^92(p46)^ This was due, in part, to the need to work part-time or to take sick leaves and presenteeism.^104,105^ Some felt that they were disappointing their colleagues or employers because they were not working normally, which contributed to their senses of stress and dispensability.^34,99^ Some subjects described still having ambition or motivation to live up to their, their colleagues’, or their employers’ expectations: “I don’t want to say to my manager that I can’t manage this, that I need some help, so I try and I clench my teeth and I ache all over … but I do the job and when I come home I collapse and take strong medication for the night to manage the next day.”^99(p103)^ Subjects reported a sense of grief over feeling as though they were not working up to their full potential.^116^ For example, some acknowledged that they were not as productive as they had been when they were well and they no longer were able to work overtime or take on extra shifts as work demanded.^92^ The unpredictability of their symptoms contributed to a sense of unfulfilled potential: “[Fibromyalgia] literally took me out of the workforce. For years I didn’t work. Something as simple as say, go be a DJ. I can’t go be a DJ. Because every day you gotta be there at a certain time. I cannot guarantee you that I am going to be there.”^92(p31)^ As a result, subjects dealt with their unfulfilled work potential issues in a number of ways: some coped as best as they could, others relied on the support of colleagues, and others left work either temporarily in the form of annual or sick leave or stopped working altogether.^34^

The loss of work-related economic benefits was a concern to subjects. The relative importance of employment income was, of course, dependent upon the subjects’ broader financial picture, including whether there were other family members who were employed.^36,89^ Thus, Liedberg and Henriksson^4^ found that some subjects could afford to decrease the amount they worked in order to accommodate the effects of their clinical symptoms, whereas others, such as those who lived on their own, felt obliged to continue working despite their symptoms. Söderberg et al.^107^ explored the perspective of spouses on this issue; they experienced greater responsibility for income generation and described greater economic difficulties associated with the loss of their partner’s income.^107^ Economic effects of fibromyalgia on subjects’ work experiences was also relevant in terms of its effects on their career trajectories. This was dependent upon the job’s characteristics. For example, some subjects found that their career advancement was not particularly affected by their symptoms because they were not employed in jobs with much physical activity.^92^ Others, however, found that their opportunities for employment advancement had become more limited. As one subject put it: “In a short sentence, [fibromyalgia] has stolen my life … [t]he life that I thought I was gonna have. The life that I had planned on having is gone.”^92(p35)^ Another subject described more specifically the career opportunities that were lost: “I was on the fast track before I came down with [fibromyalgia]. I was on the fast track with the company, I was being looked at as a possible senior VP position, and, you know, my career has pretty much stagnated at this point. The health issues, the constant doctor’s appointments, the exhaustion has definitely affected the production level.”^92(p36)^

Given the importance of work to well-being and identity, it is not surprising that a common topic of subjects’ interviews was the deleterious effects that fibromyalgia symptoms had on their sense of identity. Asbring^89^ discusses the manner in which a chronic illness can cause a “biographical disruption” in people’s lives, which happens in the context of them not being able to perform their usual activities, including work, or to interact with others, such as work colleagues, as they had in the past.^89(p318)^ Thus, fibromyalgia can affect identity because, as Asbring puts it, “activities which previously could be engaged in are more difficult or totally impossible because of the illness, and there is a disruption between the individual’s definition of herself with respect to the past, the present and the anticipated future.”^89(p313)^

Study participants described work as having inherent value.^4,34,81^ In Palstam et al.’s study,^35^ some subjects continued to work despite its associated hardships because of its importance to their sense of self and because it was satisfying to them. For example, work was described as being “stimulating” and associated with “continuous development.”^35(p1624)^ It gave them a sense of being “needed” and “appreciated.”^35(p1624)^ In Liedberg and Henriksson’s study, work gave subjects the sense as though they were “a part of society”: “We are taught that without work we are not worth anything … [i]t is as if I exist when I get out [to work] … you are confirmed in some way … otherwise you are nobody, just a burden on others.”^4(p268)^ Leaving the workforce can give patients the sense that they are now “outsiders.”^98(p448)^ As another subject stated:

There is nothing as terrible as not having a job. You have no social life, you get very isolated and … life does not feel meaningful, so to speak. Something is missing … it’s not natural not to work … it’s unnatural. That’s the meaning of life … just to feel that you are useful … that you are needed in some way.^4(p268)^

Some of the subjects in Juuso et al.’s study described themselves as “workaholics” and work for them had been “the essence of their lives.”^96(p73)^ Work was associated with having self-worth and the inability to work decreased self-esteem.^96^ To be told by employers that they could no longer work because of the way in which their fibromyalgia symptoms precluded this was associated with a “sense of betrayal” for some subjects; their previous identity as valued employees was no longer valid and they felt treated “just like a number.”^34(p567)^

Beyond meaning, work also provided “structure.”^35^ To occupy themselves with work also provided an opportunity for distraction from the symptoms of fibromyalgia.^35,96^ As one subject said:

When I sit at home in pain, I get so isolated in my head by my pain. Getting out of the house, being at work among healthy people is enough to be able to forget that I have a problem. There (at work) I suddenly become a healthy person again. I can throw this crap behind me and be healthy for a while.^35(p1624)^

This sense of health from work was recapitulated by Löfgren et al., who noted that patients with fibromyalgia “are more satisfied with their life situation and report better health status then non-working fellow-sufferers.”^98(p448)^

Asbring^89^ describes a transformation of identity occurring among the patients in her study who were no longer able to work due to their fibromyalgia symptoms. Some strove to retain their former identity as much as possible, whereas others described having a new identity that was described in terms of otherness, “as another self lying outside the normal self, living its own life.”^89(p315)^ The more active the subjects’ lives had been prior to their diagnosis, the more difficult the readjustment of identity was.^89^

For some subjects who were unable to return to work because of their illness, grieving and coming to terms with what had been lost in their lives was still possible. Löfgren et al. described how this was possible for some of the subjects in their study who had gone through a rehabilitation program through the Karolinska Hospital:

To reach positive ways of looking upon oneself, and constructive ways of dealing with problems, the informants had to work through the loss of their former self and their former body. Living with [fibromyalgia] meant difficult feelings of disappointment with life, self-blame at not being able-bodied, sadness, despair and exhaustion, regret for the parts of life that no longer could be lived. They reached a turning point through grieving and accepting the situation, and a new way to manage life could develop.^98(p452)^

### Fibromyalgia Strains Relations at Work

Beyond the individual benefits of working in terms of issues of self-worth and economic benefits to the household, employment for patients with fibromyalgia also served an important role in maintaining relationships with others. Subjects described one of the benefits of working as being the opportunity to socialize with others and build their social networks, which in turn also enhanced their satisfaction with their work itself.^4^ Subjects in Palstam et al.’s^35^ study described feeling less lonely because they were able to continue working. This was echoed by subjects in the study by Liedberg and Henriksson.^4^ For example, as one 26-year-old woman stated:

To me, work means having social contacts and getting back into society again. Not living on the outside and looking in … [s]o for me it is almost only positive … to get out into the labor market and meet people, even if you are in pain, and even if it occasionally is an infernal ache. That does not matter … because it is worth it.^4(p268)^

Having the support of supervisors and colleagues was an important contributor to subjects’ abilities to continue working and enjoying their work experiences. For example, one of the benefits of colleague support was that their assistance enabled subjects to continue working because they “receiv[ed] help with heavy work tasks, [were] relieved of an excessive workload and receiv[ed] understanding and acceptance at the workplace.”^35(p1627)^ One of the subjects in Mannerkorpi and Gard’s study described having more confidence to continue working because of such support: “I have a good situation, I can call my supervisor and say I have to come to work a little late today, I can’t manage to get out of bed just now. [The supervisor said] ‘No, come when you want!’”^99(p102)^ Unfortunately, not all subject were the recipients of such support; indeed, in Löfgren et al.’s study,^98^ it was more common for subjects not to have the support of colleagues. One of the key differences identified between women who were working with fibromyalgia and those who were not was that women who were no longer working had experienced less social support at work compared to women who were still working.^112^ A common theme among the reviewed studies was that once individuals stopped working, they lost their social networks.^4^ A questionnaire study has suggested that these social networks are particularly important to the development of social capital among patients with fibromyalgia.^109^

Among the reasons why obtaining social support from colleagues in the workplace has been reported to be challenging by subjects has been the issue of stigma related to the fibromyalgia diagnosis. Fibromyalgia has been described as an “invisible” illness because its symptoms may not necessarily be manifest with particular physical signs or symptoms.^93^ Toye et al. describe their subjects as being involved in a “battle for legitimacy”^34(p569)^ at work in terms of having to prove to coworkers and supervisors that their symptoms affected their ability to work in a real and legitimate manner. Stigmatization was a theme commonly expressed by the subjects in the studies by Dennis et al.^91^ and Juuso et al.^96^ Coworkers were described as being unsympathetic; they thought that subjects were malingering so that they would not have to do as much work and resented having to potentially cover for their colleagues with fibromyalgia because there was a lack of trust in the validity or reality of their illness.^91,96^ In order to legitimize their illness and its effects on their ability to work, subjects described being forced to seek health care, often at the expense of being able to stay at work.^34^

Given this, it is not surprising, then, that a common issue raised by subjects in the qualitative studies was the struggle that they described relating to whether to disclose their illness to their colleagues. Some subjects found disclosure of their condition at work to be “empowering” because it meant the opportunity to clarify for their colleagues that there was a reason for their particular work habits.^92(p53)^ Other subjects found that disclosure only made them feel devalued:

No, I prefer not to say so much, I was mocked at work when I had a hard day and had a lot of pain under my feet. It’s like walking on glass actually and I had taken with me a paper about the pain so I could explain and then she came [the boss] and saw the paper and mocked me about it and that’s why I’ve backed off and I don’t want to tell anyone about the pain.^95(p7186)^

Oldfield et al.^100^ focused their study on the issues that women with fibromyalgia have with regards to disclosure of their medical condition in their workplaces. Among the themes that their semistructured interviews uncovered included those related to the scrutiny that subjects exposed themselves to when they disclosed their illness. As discussed above in the context of fibromyalgia-associated stigma, disclosure sometime had the effect of further alienating subjects from their coworkers. Alternatively, disclosure was a beneficial act if coworkers responded with empathy. Some subjects selectively divulged aspects of their condition; for example, some divulged only certain aspects of the condition itself, or divulged information only to selected individuals. Divulging of information occurred for a few reasons, including the hope to preempt colleagues for resenting them for certain accommodations that they may have been afforded and to provide an explanation for their inability to perform certain jobs. In some cases, the nature of the illness itself was not described fully. For example, one subject described having a “really bad knee” as opposed to disclosing that she had fibromyalgia because it was thought that this would be “easier for them [coworkers] to understand” and would be more believable than a fibromyalgia diagnosis.^100(p1449)^ Another topic related to disclosure that was addressed related to the risks of disclosure to supervisors in terms of future job prospects. Disclosure to coworkers was expressed by some subjects as easier than disclosure to supervisors because coworkers were perceived to have less power in terms of potentially limiting work.

### Fibromyalgia Symptoms Have Repercussions on Work Ability

Among the repercussions of the challenges that individuals with fibromyalgia have in the workplace has been a sense of embitterment. This topic was exclusively studied by Blom et al.^108^ Their questionnaire study revealed that 16% of patients met the criteria of clinical embitterment, as assessed by the Bern Embitterment Inventory, and was associated with the sense of helplessness with regards to their fibromyalgia symptoms and invalidation of their experience in the workplace.

Subjects’ experiences in the workplace and in the home setting influenced each other. For example, subjects described feeling exhausted after a day’s work and appreciated the support of family members once at home.^99^ Some subjects described a redistribution of household-related tasks that occurred to facilitate subjects’ continued employment.^4^ Subjects expressed guilt that they were unable to help their family members as much as they would have liked.^99^ Being able to continue working was much more challenging, or impossible, for subjects who did not have such family support.^4,99^

### Patients Consider a Variety of Solutions to Remain Employed

Subjects described developing or having access to a number of strategies to facilitate their continued employment. For example, some had the flexibility of their work schedule to change the order in which certain tasks had to be performed during the day; tasks that were more challenging to accomplish could be performed when fibromyalgia symptoms permitted.^98^ Others physically modified their workspaces to improve ergonomics. Thus, one office worker used a stand-up desk and a daycare worker dressed children one at a time or had them climb onto a nursing table themselves as opposed to picking them up herself.^98^ Being able to modify certain aspects of their jobs was easier for some subjects compared to others. Flexibility over certain aspects of the jobs also enhanced subjects’ abilities to remain employed. Some subjects were able to use a cell phone instead of being constrained to a desk using a landline.^34^ Others had the ability to delegate certain aspects of their jobs and share job tasks.^34^ Half of the subjects in Rakovski et al.’s study^5^ had flexible job hours and liberty to modify the type of work that they did. Some subjects were able to take breaks during the day as needed and so control their work schedules.^35,90^ Subjects described reducing their hours, reducing the number of days of the week that they work, or taking sick leave.^4,99^ Others changed their out-of-work schedule such that they did not participate in social events until the weekends.^98^ The ability for such solutions was dependent upon the types of jobs that these subjects had; thus, subjects in competitive job situations felt less able to modify their work.^4^ Subjects who were working in service-related occupations had less control over their work environment compared to subjects who were managers or had “white collar jobs.”^4,93^ This ability to control the work environment positively correlated with job satisfaction.^93^

## Discussion

The literature surrounding employment and workplace issues for patients with fibromyalgia has included qualitative and questionnaire studies. Although they offer a lower level of evidence, synthesizing their results has a role to play in terms of providing ideas to guide best practice. Qualitative studies have examined subjects’ attitudes, feelings, and opinions regarding the aspects of fibromyalgia that interfere with their abilities to work and have outlined the ways in which subjects cope with their symptoms at work. Questionnaire studies have provided further information about the manner in which fibromyalgia symptoms have affected employment for patients with fibromyalgia, such as by outlining the factors that patients find contribute to their ability or inability to work. Overall, a review of 37 studies found that (1) fibromyalgia symptoms are severe enough to adversely affect patients’ abilities to work; (2) the development of social networks via working is a means by which patients with fibromyalgia maintain their function and social support is an important facilitator of continued employment; (3) embitterment and a transformation of life outside of work are reported consequences of trying to manage fibromyalgia and yet continue working; and (4) patients with fibromyalgia develop a number of strategies to try to balance their illness symptoms with their desire or need to work.

There are strengths to the research methods employed by the various studies. For the questions that these studies were trying to answer, a qualitative approach that looked in-depth at either employed or unemployed patients’ experiences provided a rich source of data. The advantage of this approach is that it allows elucidation of “data from the experiences of the workers [or patients] themselves, thus opening up the stud[ies] to authentic themes, independent from prevailing constructs.”^28(p127)^ Such an approach also allows researchers a great degree of flexibility in gathering data.^117^ It also means that data can potentially be gathered “without the theoretical constraints imposed by the interviewer”^28(p127)^ and this thus “permits a better understanding of ‘the quality and texture of experience’, ‘how people make sense of the world’, and how they experience and manage events or conditions.”^118(p448)^ This was certainly the case in the studies outlined in Table 1.

Done well, the grounded theory approach to data analysis used by a number of the studies offers a legitimate and appealing manner in which to “understand the process by which actors construct meaning out of intersubjective experience.”^119(p634)^ Ruppel and Mey summarized the theory as “enabl[ing] a systematic analysis on the basis of clearly defined analytic steps, while at the same time being sufficiently open to provide researchers with room for maneuver in its applications.”^120(p175)^ Oliver described grounded theory as using induction in order to allow for the “systematic development of theory.”^121(p376)^ The ultimate objective is “a description of the relationships between conceptual categories and their synthesis into a theory explaining the maximum amount of variation within the issue of concern.”^121(p377)^ McCann and Clark^122–124^ described seven features of grounded theory: (1) theoretical sensitivity (in which researchers must have some knowledge of the topic under investigation so that the can “give meaning to the data”^122(p10)^; (2) theoretical sampling (which has also been described as purposeful sampling); (3) constant comparative analysis; (4) coding and categorizing data; (5) the formulation of theoretical memos and diagrams; (6) using literature as a source of data; and (7) integration of theory. In addition to thematic analyses, other studies employed a phenomenological–hermeneutic approach to analyzing the qualitative data.

Nevertheless, the qualitative approach to this topic has a number of weaknesses. Firstly, the sample sizes of subjects in many of the included studies, particularly the qualitative studies that relied on interviews with patients with fibromyalgia, were small (Table 1). The concern is that the results from small samples may not be representative of the population of patients with fibromyalgia and so are not generalizable. Concerns are raised regarding the generalizability of the results of questionnaire studies, as well, given that some of the response rates were low. For example, the response rates in the studies by Blom et al.^108^ and Guymer et al.^3^ were 48% and 17.4%, respectively. Secondly, the manner in which subjects were selected in some studies introduced the possibility of selection bias. For example, some patients were recruited through newspapers, websites, conferences, and advocacy groups.^91,92,109,111^ There may be certain characteristics of patients who are more apt to volunteer for these sorts of studies that differentiate them from patients who do not in ways that are relevant to attitudes toward workplace accommodations. Other subjects were not recruited in a random fashion but instead were purposely chosen because it was thought that they would represent certain perspectives. Thus, some studies only included women.^88,90,95,96^ Another only recruited from a clinic in an affluent neighborhood of Boston,^88^ whereas others only recruited from hospital primary care^110^ or specialty clinics.^88,95,96^ This means that these samples may have been more homogenous than would be expected compared to a random sample of patients with fibromyalgia. Depending on the location from which the patients were recruited, they may have been either less or more ill compared to a randomly selected population of patients with fibromyalgia. Thirdly, the results of the qualitative studies may have been influenced by recall bias, because patients with fibromyalgia were asked to reflect upon their past experiences as they related to work. Sallinen et al.^104^ and Choy et al.^10^ acknowledged that it is possible that memories may have been distorted. Fourth, the capturing of data from one-time interviews means that perspectives and attitudes are only captured at one particular time point. It is possible that longitudinal studies may provide additional or even different data, given the variable nature of fibromyalgia symptoms. Gauer^93^ argued that point because he noted that responses to interview questions may have been different had the patients been having a particularly symptomatic or asymptomatic day. Fifth, one study relied on patients to self-identify as having fibromyalgia rather than relying on physician verification.^112^ Given the results of a recent study that suggested that as few as 25% of patients who self-identify as having fibromyalgia actually meet the clinical criteria for diagnosis means that the results of that study may not be generalizable to patients with fibromyalgia.^86^ Another weakness to these qualitative studies is that the overall strength of evidence of such studies is low.

Despite the attractive nature of grounded theory as a way to investigate research topics from a qualitative perspective, it, too, has been subject to a number of criticisms. For example, McCann and Clark suggested that the theory demands that a researcher be “simultaneously objective and subjective” and that this creates an internal “tension.”^123(p20)^ Thus, though researchers should not begin a study with preconceived hypotheses or ideas about what the data will show, they also cannot begin the study without having at least some background knowledge that allows them to determine which data are important and which are not. They additionally indicated that the use of purposeful sampling has the potential to make the project “lack conceptual depth” because the researchers have complete control over the study participants.^123(p20)^ As a result, though researchers may strive to obtain a diversity of opinions from research subjects, their attempts to obtain that diversity may actually undermine it if their sampling methods are not appropriate.

Antao et al.^33^ reviewed work sustainability in the context of illness-associated chronic pain, and their discussion of the barriers that affect patients’ abilities to continue working despite pain is relevant to the situation affecting patients with fibromyalgia. Their discussion occurs in the context of identifying different levels at which barriers can be identified, such as at the level of the individual patient (“micro”), the level of the workplace (“meso”), and at a societal sociopolitical level (“macro”).^33^ The ideas that they present can be applied to the fibromyalgia context. At the micro level, barriers to patients with fibromyalgia continuing employment include the loss of control that their chronic pain condition imparts. The symptoms themselves, as well as their variable severity and characteristics, make it difficult for patients to cope with the pain and continue working. The symptoms also may contribute to difficulties that patients may encounter with certain physical or cognitive aspects of their work. Altogether, clinical symptoms coupled with ongoing health care provision needs can socially isolate patients with fibromyalgia in their workplace. At the meso level, barriers to continued employment include the incongruity of workplace or employment demands and patients’ abilities. Colleagues’ and supervisors’ lack of knowledge about fibromyalgia may contribute to the lack of provision of appropriate accommodations. At the macro level, barriers to ongoing employment include social systems that do not provide adequate social supports or benefits and the persistence of stigma regarding the fibromyalgia diagnosis.

Potential ways to foster the continued employment of patients with fibromyalgia may be similarly considered in this multilayered context.^33^ At the micro level, patients could receive education about their employment rights, such as those that relate to reasonable accommodation requests, pain self-management strategies, and further skill development to facilitate potential alternative career options. Palstam et al.^35^ additionally recommended the promotion of the development of work coping skills among patients. Physical strategies could include the use of assistive devices. Advocacy for greater flexibility in terms of work schedules and for job accommodations could also prove useful for patients.

At the meso level, education strategies are also potentially useful as they relate to providing coworkers and supervisors with greater information about the reality of the challenges that patients with fibromyalgia experience in the workplace to dispel stigma and myths and facilitate greater social support. In Gauer’s study, “without exception, participants expressed belief that their work situation would improve if their coworkers understood more about [fibromyalgia], including its tangible effects on their productivity and motivation, and how [fibromyalgia] is a legitimate illness that they suffer, not an excuse to be lazy.”^93(p129)^ Physical strategies can help as well, such as by modifying work environments in a manner that lessens the effects of work on patients’ symptoms. Some examples of ways to accomplish this include allowing workers the opportunity to vary their work positions and tasks and to take short breaks.^99^ The provision of accommodations, such as allowing patients greater flexibility in terms of their work hours and time for them to attend medical appointments, are also potential solutions. It has been suggested as well that “heavy physical tasks, frequent carrying and lifting, static movements, dynamic repetitive work and eccentric muscle work need to be limited.”^99(p101),116^ These solutions make sense according to the job strain model, as originally described by Karasek^125^ and reviewed by Gauer.^93^ Here, job strain is depicted as being related to job demand and decision control. Having low job demands with high decision control predicts low job strain, whereas having high job demands and low decision control predicts high job strain. Mannerkorpi and Gard^99^ also found that patients sought a balance between job demands and their individual abilities to cope with their symptoms. These solutions have also been shown to support continued work presence by women with fibromyalgia.^116^ In a Swedish study of 176 patients, of whom 15% were working full-time and 35% were working part-time, the ability to influence the performance of work tasks facilitated continued employment.^116^

At the macro level, patients have identified that having access to social support systems, such as sick leave, facilitates their attempts to manage their illness while still remain employed. Such was argued by patients interviewed in the study by Palstam et al.; having the opportunity to legitimately take time away from work to deal with symptoms allowed the creation of a “sustainable life with balanced work demands.”^35(p1627)^ Policies that counter harassment and discrimination have been described for patients with multiple sclerosis or human immunodeficiency virus and are relevant for patients with fibromyalgia as well.^33,126^ Additionally, social support systems need to recognize that the work ability for patients with fibromyalgia, or patients with chronic pain in general, is not necessarily dichotomous in terms of being entirely possible or entirely impossible. Rather, patients have varying abilities to engage in work depending upon the severity of their condition. As a result, Antao et al.^33^ recommended that government programs be flexible in terms of their requirements for patients to meet the criteria for employment insurance benefits, sick leave benefits, social assistance, and health benefits in a way that supports patients’ attempts to remain employed or go back to work. For example, patients in Palstam et al.’s^35^ study found that Swedish Social Insurance Agency support allowed them to reduce their work hours such that they could work over the long term.

Part of the solution can also be argued to lie with changes in the manner in which members of the health care profession interact with patients with fibromyalgia. Asbring^89^ wrote that patients with chronic illness, especially those that do not manifest with physically obvious abnormalities or with objective clinical findings, find themselves at risk of not having their symptoms being taken seriously. Indeed, the very existence of the condition may even be questioned.^89^

In the mid-20th century, fibromyalgia was “thought to be a hysterical manifestation of psychogenic rheumatism.”^88(p18)^ It was considered to be a psychological illness and its existence as a veritable clinical entity was questioned.^89^ Recently, a variety of clinical practice guidelines have been developed and published.^127–129^ A recent multicenter study performed in eight countries found a deficiency of knowledge among physicians surveyed.^10^ For example, 45% of physicians were not aware of the American College of Rheumatology (ACR) fibromyalgia diagnostic criteria and 11% and 42% considered making a diagnosis of fibromyalgia to be “very difficult” and “somewhat difficult,” respectively.^10^ It is therefore not surprising that patients with fibromyalgia were only diagnosed 2.3 years after developing symptoms and after seeing over three different physicians.^10^ Patients interviewed in the studies corroborated the idea that there was often a lack of knowledge about fibromyalgia in the medical community:

Primary doctors don’t seem to know too much about the condition. My first primary doctor was the one who sent me to the rheumatologist for treatment … and my new one seems pretty clueless. He made some copies of some information that a rheumatologist gave him to help him be able to treat fibromyalgia on his own, but that seemed to be the extent of his knowledge. … I feel like they all want to push medications on me. Now that I have chosen not to go on medications, my rheumatologist seems to not know what to do with me.^91(p773)^

A common theme among studies examining fibromyalgia and employment included patients encountering stigma from health care professionals.^108^ Physicians who were interviewed regarding the presentation of patients with fibromyalgia commented that

there was a discrepancy between how persons with … fibromyalgia represented themselves in the encounter with the physician and how a sick person … is expected to look and behave … What is very characteristic is that they look so healthy, move very casually and that there is a certain gulf between the pain they bear and how they behave.^52(p714)^

Åsbring and Närvänen noted that there was often a “judgmental assessment of illness” that occurred^52(p717)^; this may have been related to issues of secondary gain being raised in physicians’ minds.^60^ In a study by Schwenk et al.,^130^ patients with complex medical problems, like fibromyalgia, were more likely to be regarded as “difficult” to deal with. Rather than getting the sense that their fibromyalgia symptoms were being taken seriously, patients described feeling as though their symptoms were “psychologized” such that they were made to believe that their symptoms were not real and their morality was questioned.^88(p28)^ Acker noted that “doctors’ explicit and implicit views about affected patients’ perception of themselves in relation to their diagnosis”^88(p58)^: “I think I got stamped as a psych case like right from the beginning and it’s like I was carrying the scarlet letter with me wherever I go.”^88(p44)^ Patients felt reluctant to see their physicians; as one patient stated, “I got nervous that they’re going to say … this chick is a nut case and she’s just a hypochondriac. Same complaints.”^88(p51)^ As another stated, “With fibromyalgia a lot of the pain changes and then you’re thinking oh my gosh, now I’m going to go in there and tell them that my left leg hurts, last week it was my right leg … and I’m thinking I’m not telling them this, this is insane.”^88(p51)^ In Sallinen et al.’s study, patients with fibromyalgia had been told by health care professionals that they had a “mental health problem”: “You didn’t really want to talk about it, ’cause you would be seen as one of those who just think they are ill … they let you understand that it doesn’t really exist … that is why it was difficult to seek help … they thought it was all in your head.”^104(p21)^ As a result, patients experienced “shame,” “anxiety,” and “self-doubt” as a result of interactions with physicians.^88(p28)^ Because of stigma, patients found that their interactions with physicians could be unpredictable in terms of the manner in which their complaints would be received.^91^ Indeed, a survey found that 38% of patients with fibromyalgia delayed their presentation to physicians because of concerns that “they would not be taken seriously.”^10(p104)^ Patients were frustrated when their physicians told them “You’re OK, that’s all right, you’re normal” despite the persistence of their symptoms.^88(p42)^ As Acker pointed out, the broader implications of stigma held within the medical community become clear: “Societal stigma is only increased when the medical community itself does not give credence to [fibromyalgia] patients’ subjective reports of their symptoms and mirrors the view that without objective physical findings, the patient must be malingering or suffering from a purely psychological condition.”^88(p1)^

A variety of health care provider–related solutions are possible. Firstly, thinking broadly, there are recommendations for health care professionals to approach chronic pain conditions such as fibromyalgia with a biopsychosocial approach to management.^88^ This means recognizing the way in which patients’ symptoms affect different aspects of their lives, such as their interactions with others and their ability to work, and how the varied facets of their lives also influence the way in which their chronic pain problems manifests.^60^ Acker suggested that one of the benefits of such an approach is that it promotes a “collaborative,” “positive,” and “empathetic” relationship between patients and their physicians.^88(p55)^ This also promotes a more individualized approach to treatment, which is important if the view of Dennis et al. is considered; they described fibromyalgia as “autobiographical” in that, for patients, the condition can be viewed “as something happening to them” and hence “the tendency to think of illness as being about them.”^91(p778)^ Secondly, physicians can recognize fibromyalgia for the chronic pain disorder that it is and help combat the stigma surrounding its diagnosis. As one patient in Acker’s study found, “For the first time I heard a doctor say … “You were not crazy; you weren’t … you’ve been going through so much’ and I think just getting that, that affirmation … it would at least get you through a little bit.”^88(p45)^ Lastly, physicians need to play a more substantive and collaborative role in discussions regarding patients’ employment issues. Toye et al.^34^ argued that, to date, the role of the physician has been to verify that an individual has an illness and issue sick notes. Much of the advice provided is vague; for example, patients were recommended to “take care” at work.^34(p569)^ Patients suffer because they find the need to take time from work to prove that their symptoms legitimately affect their ability to work, but this “battle for legitimacy … does not necessarily facilitate a return to work.”^34(p569)^ They think that there needs to be better communication between individuals and their physicians and employers so that strategies to foster continued employment can be discussed.^34^ For example, Palstam et al.^35^ suggested that physicians should provide greater support to patients in terms of helping them to advocate for more ergonomic workplaces. Toye et al. suggested that physicians write “fit notes” that give more specific advice about the factors that would enhance their patients’ ability to remain employed or return to work, such as what work duties should be modified and how.^34(p570)^ Given that fibromyalgia is estimated to affect up to 8% of the general population and twice as many women as men,^1^ health care providers’ promotion of such solutions has the potential to particularly help a significant number of female patients with their employment goals.
